# Contribution of Free-Text Comments to the Burden of Documentation: Assessment and Analysis of Vital Sign Comments in Flowsheets

**DOI:** 10.2196/22806

**Published:** 2021-03-04

**Authors:** Zhijun Yin, Yongtai Liu, Allison B McCoy, Bradley A Malin, Patricia R Sengstack

**Affiliations:** 1 Department of Biomedical Informatics Vanderbilt University Medical Center Nashville, TN United States; 2 Department of Electrical Engineering and Computer Science Vanderbilt University Nashville, TN United States; 3 Department of Biostatistics Vanderbilt University Medical Center Nashville, TN United States; 4 School of Nursing Vanderbilt University Nashville, TN United States

**Keywords:** electronic health system, documentation burden, flowsheets, content analysis, vital sign comments, free text

## Abstract

**Background:**

Documentation burden is a common problem with modern electronic health record (EHR) systems. To reduce this burden, various recording methods (eg, voice recorders or motion sensors) have been proposed. However, these solutions are in an early prototype phase and are unlikely to transition into practice in the near future. A more pragmatic alternative is to directly modify the implementation of the existing functionalities of an EHR system.

**Objective:**

This study aims to assess the nature of free-text comments entered into EHR flowsheets that supplement quantitative vital sign values and examine opportunities to simplify functionality and reduce documentation burden.

**Methods:**

We evaluated 209,055 vital sign comments in flowsheets that were generated in the Epic EHR system at the Vanderbilt University Medical Center in 2018. We applied topic modeling, as well as the natural language processing Clinical Language Annotation, Modeling, and Processing software system, to extract generally discussed topics and detailed medical terms (expressed as probability distribution) to investigate the stories communicated in these comments.

**Results:**

Our analysis showed that 63.33% (6053/9557) of the users who entered vital signs made at least one free-text comment in vital sign flowsheet entries. The user roles that were most likely to compose comments were registered nurse, technician, and licensed nurse. The most frequently identified topics were the notification of a result to health care providers (0.347), the context of a measurement (0.307), and an inability to obtain a vital sign (0.224). There were 4187 unique medical terms that were extracted from 46,029 (0.220) comments, including many symptom-related terms such as “pain,” “upset,” “dizziness,” “coughing,” “anxiety,” “distress,” and “fever” and drug-related terms such as “tylenol,” “anesthesia,” “cannula,” “oxygen,” “motrin,” “rituxan,” and “labetalol.”

**Conclusions:**

Considering that flowsheet comments are generally not displayed or automatically pulled into any clinical notes, our findings suggest that the flowsheet comment functionality can be simplified (eg, via structured response fields instead of a text input dialog) to reduce health care provider effort. Moreover, rich and clinically important medical terms such as medications and symptoms should be explicitly recorded in clinical notes for better visibility.

## Introduction

### Background and Motivations

Electronic health record (EHR) systems have been widely adopted in clinical settings over the past decade [1]. These systems have provided many benefits that include, but are not limited to, improving quality of care [2], reducing prescription errors [3], and facilitating biomedical research [4]. Despite such benefits, documentation burden has been recognized as a negative artifact of adopting EHR systems. For instance, it was shown that primary care clinicians spent more than 50% of their time in front of an EHR system, thus reducing their time in interactions with patients [5]. In another study, it was reported that ophthalmologists spent approximately 3.7 hours per day using EHRs [6]. It has also been shown that nurses, one of the largest EHR system users, enter approximately 640 flowsheet data entries during a 12-hour shift, nearly one data point every minute in acute care [7]. In addition, in a web-based survey conducted in the Nursing Quality and Care Forum, 78% of the participants confirmed that documentation in EHRs is time-consuming and difficult to complete, and 68% suggested that such documentation contributed little value to patient care [8].

Documentation burden originates from various factors, such as the complex functionalities of EHR systems (which itself is partially due to increasingly sophisticated health care routines), increase in the amount of data being collected, and the challenge of prioritizing the information scattered in different locations in an EHR system [9]. The US Department of Health and Human Services has released strategies to reduce the burden of using health information technology (and EHRs in particular) [10], noting that the causes of documentation burden are many and complex and must be addressed on several levels by EHR vendors, regulatory agencies, insurers, and health care organizations themselves. In particular, one of the proposed strategies is to simplify documentation requirements for evaluation and management by streamlining Medicare Physician Fee Schedule final rules.

In addition to policy changes, it has been suggested that alternative recording strategies could reduce documentation burden. In one study, a smartwatch app with voice recognition was designed to help nurses record discussions during patient care, which could subsequently be uploaded to the EHR system [11,12]. A more recent study suggested that clinical documentation based on a collaborative wiki could provide opportunities to reduce documentation burden [13]. Other proposals include using artificial intelligence apps for auto-generation (eg, for treatment planning or summarization for radiation oncology [14]) or motion sensors and cameras to automatically populate EHR data (eg, in emergency care [15]). However, these approaches are limited in that most are in a prototype phase and are unlikely to be ready for implementation in the near future. Although it has been shown that using medical scribes (individuals who specialize in transcribing information during encounters into EHRs in real time) can reduce the documentation burden for physicians [16,17], scribes require a significant amount of training and clear coordination with physicians. In addition, the presence of a scribe might cause uncomfortable conversations during physician-patient encounters.

An alternative solution, with the potential for an immediate effect, is to customize the functionalities of an existing EHR system. For instance, it was shown that turning off certain interruptive notifications could help reduce EHR alert fatigue [18,19]. However, before doing this, it is necessary to investigate the functionality that is going to be customized to minimize negative consequences. In addition, organizations are beginning to examine free-text comments in EHRs, particularly those found in nursing flowsheets where the intent is to provide a place for succinct and standard responses.

### Free-Text Comments in Flowsheets

**Figure 1:**
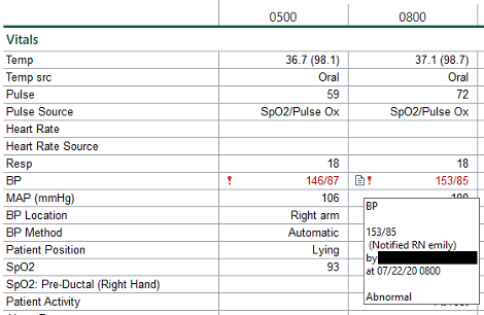
Example of a vital sign comment that is entered in the Epic system. © 2020 Epic Systems Corporation.

Flowsheets are standardized tools in EHR systems that are helpful in documenting longitudinal patient information (eg, assessments, observations, and routine care) in a grid-type format [20]. In each flowsheet entry, a health care provider can enter values into a cell from provided lists or types in numerical values such as blood pressure (BP) or temperature. Additional free-text comments can be entered into a flowsheet cell, but this is not mandatory. By default, the comments (if any) are hidden behind an icon within the flowsheet entry. Health care providers can review a comment by clicking or hovering over the icon to open the comment display dialog. Figure 1 depicts a screenshot of a vital sign flowsheet with comments entered for BP. Although the flowsheet comments are optional, some health care providers find them useful and make an extra effort to provide them [21]. However, comments may introduce a documentation burden stemming from limitations in the existing EHR functionality. Given that flowsheet comments are made accessible in a nonobvious manner, we believe that their content can be leveraged to design more effective strategies for efficiently recording them.

### Research Objectives

In this study, we seek to investigate the nature of flowsheet comments and their contribution to the documentation burden. In particular, we focused on the vital signs in flowsheets and extracted all their related comments written in 2018 in Epic, the EHR system that is in use at the Vanderbilt University Medical Center (VUMC). Specifically, we investigated the following research questions (RQs):

- RQ1: How often are the free-text comments in vital sign flowsheet entries made and by whom?
- RQ2: What are the general topics communicated in these comments?
- RQ3: Are there any specific medical terms mentioned in these comments?

Investigating the first question provides insight into how often health care providers use the flowsheet comment functionality. Answering the latter 2 questions provides insight into potential improvements to this functionality in an EHR system. Without an understanding of how often this feature is used and the purpose it serves, it is difficult to identify potential improvements to reduce the need to add documentation beyond the expected and standard response in a flowsheet cell. If medical terms or concerns are being added to essentially hidden flowsheet comments, organizations can identify system usability enhancements to better capture patient issues that need to be addressed.

## Methods

### Data Preparation

In this study, we collected all vital signs and their comments (if present) that were recorded in flowsheets between January 1, 2018, and December 31, 2018, at VUMC. We focused on 5 specific vital signs that are commonly collected for routine clinical use in both the inpatient and outpatient settings: body temperature (*Temp*), *BP*, oxygen saturation (*SpO_2_*), pulse rate (*Pulse*), and respiration rate (*Resp*). For each vital sign flowsheet entry, we collected the user ID, user role, documented time, and the free-text comment entered. Our study did not involve any patients and was designated as exempt from human subject research under a VUMC Internal Review Board protocol. Multimedia Appendix 1 presents the number and percentage of other types of vital signs.

### Commenting Statistics and Temporal Trend

To investigate RQ1, for each vital sign, we captured the total number of unique users who entered at least one value in the flowsheet *(total users*), the number of unique users who made at least one comment *(users commenting*), the total number of flowsheet entries *(total entries*), the number of entries with comments *(entries with comments*), and the median number of words in the comments (*comment length*). We also showed the temporal trend by illustrating the number of comments of each vital sign that were generated weekly in 2018. In addition, we counted the number of comments per user role and ranked them based on their comment volume in descending order. We report the top-ranked roles that together generate at least 90% of all the comments.

### Topic Modeling

To gain insights into what was communicated in these comments, we had to rely on an efficient method to summarize such a large volume of free texts. Topic modeling is a computational method for discovering the latent *topics* that occur in a collection of documents. To apply this technique, we first manually cleaned the comments by replacing commonly misspelled words with canonical representations. For example, we replaced *rn* *notifed* with *rn notified.* After data cleaning, we applied latent Dirichlet allocation (LDA), specifically its implementation in the Gensim Python package (version 3.8.0), to identify topics. LDA is a common topic modeling technique in natural language processing to infer 2 distributions from a large number of documents. The first distribution describes the probability that a topic is sampled to form a document. The second distribution describes the probability that a term is sampled from a topic. We used the first distribution to determine the popular topics mentioned in vital sign comments and the second distribution to explain what a specific topic is talking about.

As LDA is an unsupervised learning method, we applied the coherence score (specifically, C_v_ with a default sliding window of 110) to optimize the number of topics. The coherence score measures the extent to which the most relevant terms (with the highest probabilities) in a topic coexist with each other in either an external data set or the documents that are applied to train topic modeling. The higher the coherence score, the more interpretable the topics. In this study, we treated each vital sign as a single document and trained LDA models for 2 to 30 topics (with a step size of 1) using all the vital sign comments. Each candidate model was trained 10 times based on a different random seed. Although the best practice is to select the model that achieves the largest coherence score [22], there may be multiple LDA models that achieve coherence scores that are not significantly different from each other. As such, we empirically chose a model from these candidates that has (1) a large average coherence score, which leads to high interpretability; (2) a small SD, which tends to generate a stable model; and (3) a small number of topics, which reduces the chance of overlaps between topics.

### Medical Terms Extraction

LDA is often effective at characterizing what is generally discussed in documents because it estimates the probabilities from term frequency (where a higher frequency indicates a larger probability). However, this technique is limited in that it is not oriented to represent detailed information, which is particularly a concern when relevant terms are rare. On the basis of this fact, we further applied Clinical Language Annotation, Modeling, and Processing (CLAMP, version 1.6.0), a toolkit that incorporates named-entity recognition algorithms, to identify medical terms with respect to 3 categories, that is, problems, treatments, and laboratory tests, as defined in CLAMP [23]. Examples of such terms are presented in the Results section. We use these medical terms to supplement the topics to gain a better understanding of the content of comments.

## Results

### Summary Statistics

During the 1-year study period (2018), there were a total of 209,055 free-text comments entered into flowsheets to further explain the data values entered for vital signs. Table 1 shows the basic statistics of the collected data. It can be seen that 63.33% (6053/9557) of the users who entered any vital signs made at least one comment in the vital sign flowsheet entries. Although BP received the second smallest number of flowsheet entries, it had the largest proportion of users who made comments and the largest number (proportion) of entries with comments. Similarly, Temp had the smallest number of total flowsheet entries but the second largest proportion of entries with comments. In contrast, Pulse and Resp had the smallest number (proportion) of users who made comments and the smallest proportion of entries with comments. Among these 5 vital signs, SpO_2_ had the largest number of vital sign entries. In total, 0.69% (209,055/29,995,045) of the vital sign entries received additional comments.

**Table 1:** Data summary statistics^a^.

Type	Users commenting, n (%)	Total users, n	Entries with comment, n (%)	Total entries, n
BP^b^	4733 (52.25)	9058	107,413 (2.04)	5,268,477
Pulse	3066 (33.76)	9081	16,814 (0.21)	7,898,699
Resp^c^	2346 (28.85)	8132	10,883 (0.18)	5,994,777
SpO_2_^d^	3614 (44.52)	8118	38,819 (0.55)	7,058,507
Temp^e^	3631 (44.08)	8238	35,124 (0.93)	3,774,585
Total	6053 (63.33)	9557	209,055 (0.69)	29,995,045

^a^Note that the users can document multiple types of vital signs. As a result, the total number of unique users is the union, as opposed to the sum, of the set users associated with a vital sign.

^b^BP: blood pressure.

^c^Resp: respiration rate.

^d^SpO_2_: oxygen saturation.

^e^Temp: body temperature.

**Figure 2:**
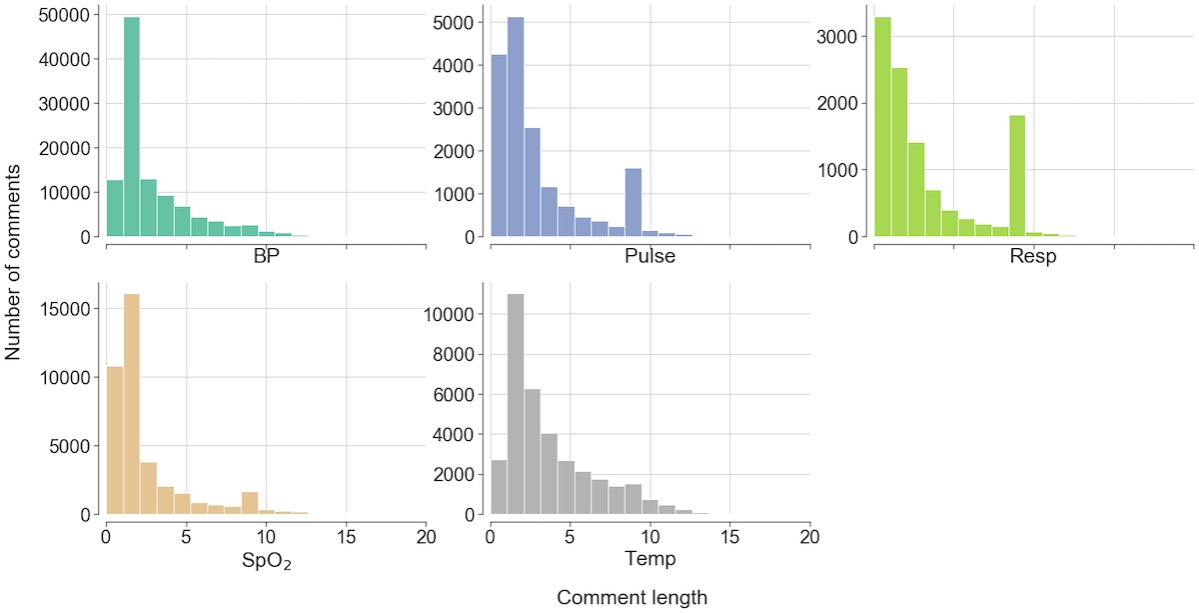
Histogram of comment length for each type of vital sign. BP: blood pressure; Pulse: pulse rate; Resp: respiration rate; SpO_2_: oxygen saturation; Temp: body temperature.

Figure 2 shows the histogram of the comment length (the number of words) for each vital sign. From the figure, it can be seen that for almost all the comments, the number of words was less than 15. Although most comments were short (with a median number of words of 2), the number of all the words used in these comments was still 697,340, owing to the large data volume.

### Comments Stratified by User Role

**Figure 3:**
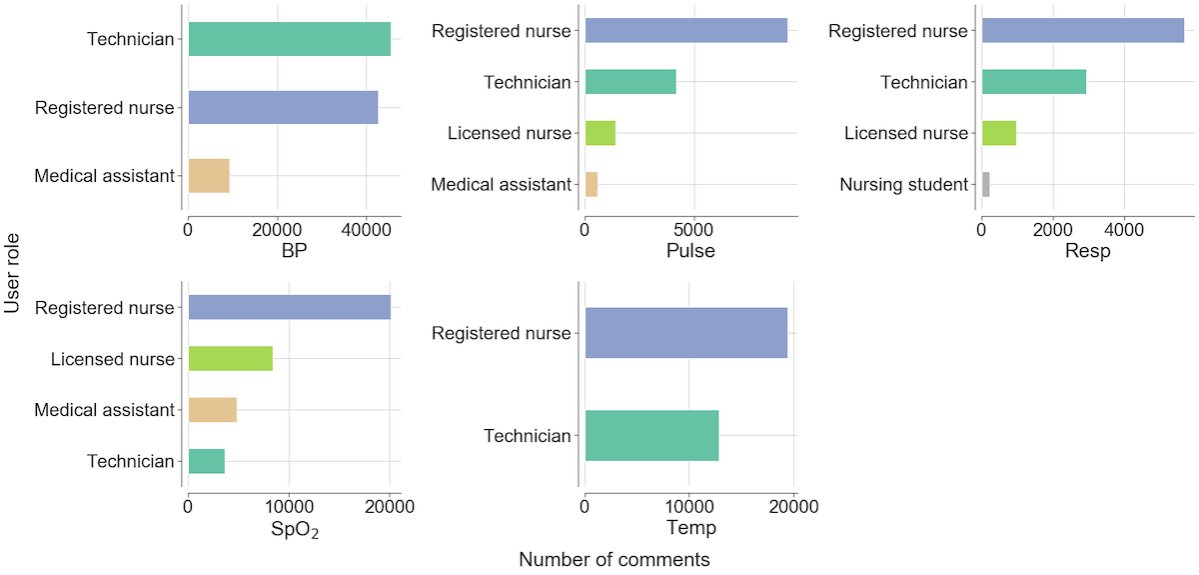
User roles with the largest number of comments per vital sign. Only the user roles that together generated at least 90% of the comments for each vital sign are shown. Registered Nurse and Technician are two user roles that generated the largest number of vital sign comments. BP: blood pressure; Pulse: pulse rate; Resp: respiration rate; SpO_2_: oxygen saturation; Temp: body temperature.

Figure 3 depicts the user roles that generated at least 90% of the comments for each vital sign. It can be seen that the user roles that were most likely to compose comments were registered nurse, technician, and licensed nurse. Although medical assistant and nursing student were among the top-ranked user roles, they generated a substantially smaller number of comments. It can also be seen that the user role registered nurse generated the largest number of comments for Temp, SpO_2_, Resp, and Pulse, whereas the user role technician generated the largest number of BP comments. The user role licensed nurse generated the second largest number of SpO_2_ comments.

**Figure 4:**
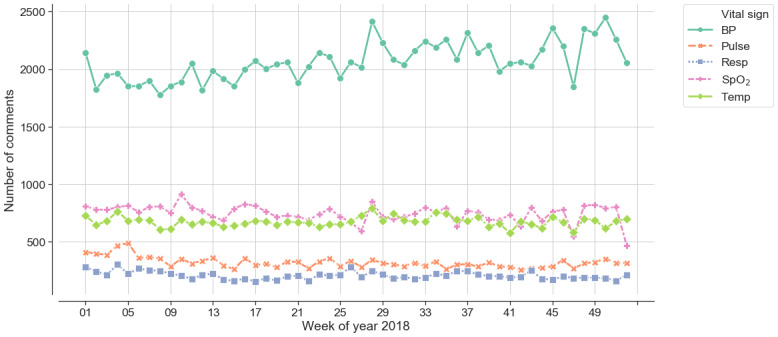
The temporal patterns of the number of comments for each type of vital sign. BP: blood pressure; Pulse: pulse rate; Resp: respiration rate; SpO_2_: oxygen saturation; Temp: body temperature.

Figure 4 shows the number of comments for each vital sign in each week in 2018. Although the number of BP comments had a slightly increasing trend, the other 4 vital signs had a relatively constant number of comments for each of the 52 weeks. This suggested that the commenting phenomenon was quite stable in this clinical setting.

### Topic Analysis

On the basis of our criteria, we empirically generated 13 topics from the flowsheet comment (see Multimedia Appendix 1 for details on how 13 topics were selected). Table 2 shows the topics, their most relevant terms, and the probability distribution. The relevant terms were selected based on their probability rank (in descending order) within a topic. For example, “notify,” “uto” (unable to obtain), “fussy,” and “move” were the most relevant terms in topic T8, indicating that a related measurement might not be obtained. Owing to the overlap between topics (eg, though a manual review), we further categorized these topics into 5 groups by examining the topic words and the associated comments (eg, examining the comments with the largest distribution of a particular topic). The percentage of each group was calculated by summing the percentage of each topic within the group. From the table, it can be seen that most comments communicated the notification of a result to health care providers (0.347), the context of a measurement (0.307), and an inability to obtain a vital sign (0.224). The other 2 topics corresponded to the measurement method (0.071) and simultaneous filling (0.051).

**Table 2:** Topics generated from vital sign comments^a^.

Label for group and topic		Most relevant words	Topic distribution (probability)		Group distribution (probability)
**Notification**				0.347	
	T1	notify, rn^b^, nurse, lie, standing, manually, informed, <NAME>, trach, <NAME>	0.106		
	T3	nurse, notify, call, high, pressure, heat, primary, hfov^c^, report, warmer	0.090		
	T9	notify, rn, nurse, elevated, <NAME>, abnormal, supine, <NAME>, <NAME>, <NAME>	0.076		
	T6	notif, team, md^d^, bedside, notifed, order, cct^e^, page, monitor, np^f^	0.075		
**Context**				0.307	
	T13	room, air	0.092		
	T4	arm, pt^g^, nc^h^, blanket, place, warm, apply, hugger, bair, baby	0.080		
	T12	post, bath, temp^i^, stand, sit, min^j^, provider, eoi^k^, inform, care	0.075		
	T7	patient, give, sleeping, tylenol, pain, med, state, liter, due, floor	0.060		
**Unable to obtain**				0.224	
	T8	patient, uto, fussy, move, crying, kicking, agitate, screaming, unit, moving	0.084		
	T2	patient, refuse, vital, sleep, asleep, time, mom, awake, defer, request	0.072		
	T11	patient, move, cry, upset, good, attempt, uto, obtain, kick, uta	0.068		
**Measure method**				0.071	
	T10	manual, cuff, bp^l^, unable, nurse, check, arm, temp, read, recheck	0.071		
**Simultaneous filing**				0.051	
	T5	datum, user, filing, simultaneous, previous, doppler, predose, cchd^m^, unnotifed, present	0.051		

^a^Topic T13 only has 2 words with positive probabilities, whereas the probability of all the other words was zero and are thus not displayed. The names in T1 and T9 are replaced with <NAME> for anonymity.

^b^rn: registered nurse.

^c^hfov: high-frequency oscillatory ventilation.

^d^md: doctor of medicine.

^e^cct: critical care team.

^f^np: nurse practitioner.

^g^pt: patient.

^h^nc: nasal cannula.

^i^temp: body temperature.

^j^min: minute.

^k^eoi: evidence of insurability.

^l^bp: blood pressure.

^m^cchd: critical congenital heart disease.

To better understand each topic, we showed the comment samples, their dominant topics (the topic with the largest probability), and topic percentages in Table 3. It should be noted that the names of health care providers mentioned in some notification-related samples were replaced with <NAME> for anonymity. Although the meanings of the samples were straightforward and clearly linked to the associated topic groups, there are still several observations that we want to highlight here. First, despite the short length, the content of comment samples contained rich information, some of which was beyond the vital signs themselves. For example, some samples in the context and notification topic groups included information regarding medications (eg, nitro paste and BP meds). This confirmed the necessity of conducting a further medical term analysis to obtain more insights into this type of information. Second, the unable to obtain topic group mainly documented why a measurement was not obtained. Finally, after a close examination of the comments with T10 (simultaneous filing topic group) as the dominant topic, we found that this topic might refer to a conflicting input of a vital sign between an automatic interface and a health care provider.

**Table 3:** Examples of comments for each topic^a^.

Topic group			Topic		Topic distribution (probability)
**Notification**					
	“Notified <NAME>, RN and <NAME>, MSN”	T1		0.182	
	“Nurse notified that pressure is high”	T3		0.139	
	“<NAME>, RN and Professor <NAME> notified”	T9		0.147	
	“Paged neuro stroke team about BP, team ordered nitro paste”	T6		0.204	
**Context**					
	“Room air, baseline oxygen sat 87% on room air preop”	T13		0.135	
	“Eating cold food and has heating pad under back/arm area”	T4		0.183	
	“Transfusion ended. No s/sx of blood tXXXransfusion reaction”	T12		0.170	
	“Patient has not taken her BP^b^ meds today”	T7		0.135	
**Unable to** **obtain**					
	“uto, patient fussy, moving”	T8		0.143	
	“Mom refused vitals, requests patient not be disturbed until wakes”	T2		0.184	
	“Patient upset, no BP obtained, multiple attempts”	T11		0.151	
**Measure** **method**					
	“Manual BP (arm measured 31 cm, used adult size cuff)”	T10		0.181	
**Simultaneous** **filing**					
	“Paced simultaneous filing. User may not have seen previous data”	T11		0.161	

^a^The dominant topic (eg, the topic with the largest probability) in each topic group is shown in the Topic column, and the corresponding probability is shown in the Topic Distribution column.

^b^BP: blood pressure.

**Figure 5:**
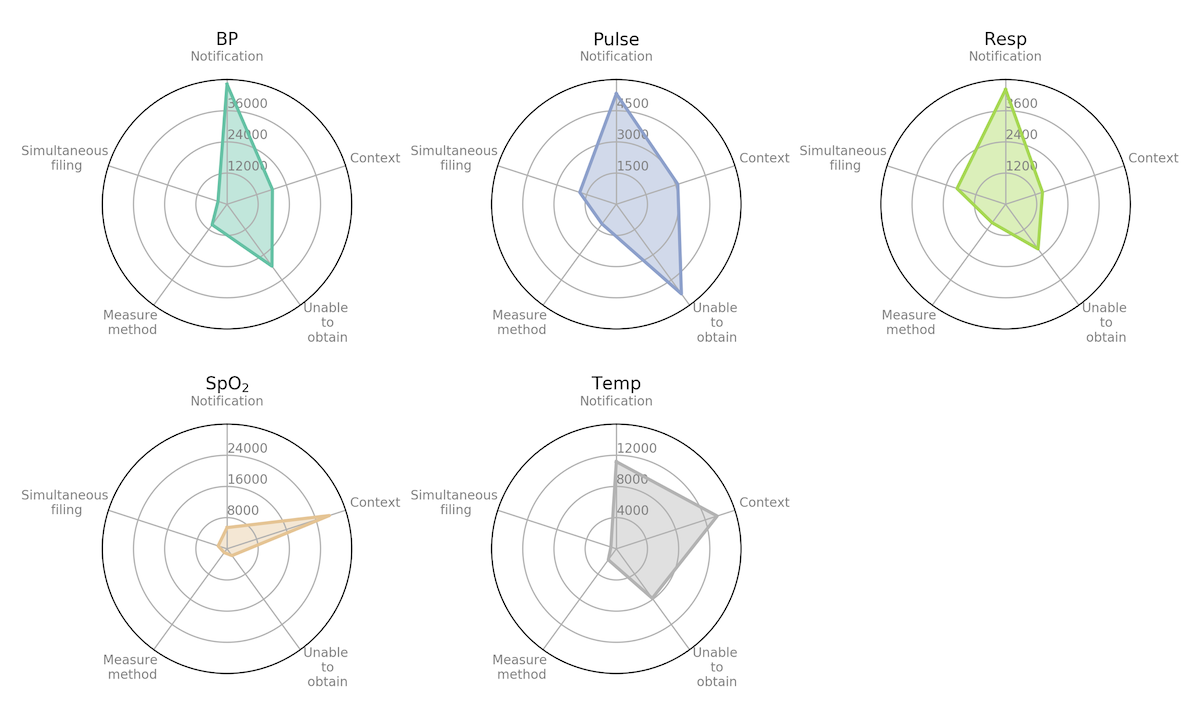
Distribution of topic groups in each vital sign. Each comment is assigned to the topic group that its dominant topic belongs to. BP: blood pressure; Pulse: pulse rate; Resp: respiration rate; SpO_2_: oxygen saturation; Temp: body temperature.

Figure 5 shows how each topic group is represented in each vital sign. Specifically, we used the dominant topic to determine which topic group a comment is assigned to and then counted the number of comments in each topic group for each vital sign. For example, all the 4 comment samples under *Notification* in Table 3 were assigned to this topic group because their dominant topics (T1, T3, T6, and T9) belonged to *Notification* (Table 2). It can be seen that some topic groups are specific to certain vital signs. For example, *notification* was the dominant topic group in BP and Resp, whereas *context* was the dominant topic group in SpO_2_. In addition, Pulse had *notification* and *unable to obtain* as dominant topic groups, whereas temp had *context* and *notification* as dominant topic groups.

### Medical Term Extraction

We applied CLAMP to generate 4187 unique medical terms or phrases that were found in 22.02% (46,029/209,055) of all vital sign comments. Of these comments, 7.66% (16,023/209,055) contained treatment-related keywords, 8.66% (18,095/209,055) contained test-related keywords, and 7.16% (14,978/209,055) contained problem-related keywords. Multimedia Appendices 2-4 depict the word clouds of the medical terms in each category. The terms *bp* and *fussy* covered 30.47% (5814/19,080) and 24.68% (3941/15,966) of all the test- and problem-related medical terms, respectively. Given their dominance, they are not reported in Multimedia Appendices 2 and 3. From the figure, we can see that test-related medical terms were mainly related to vitals, which intuitively makes sense because this study focused on vital sign comments. However, there were other tests, such as magnetic resonance imaging, which were mentioned in these comments. The problem-related terms included many symptom-related terms such as “pain,” “upset,” “dizziness,” “coughing,” “anxiety,” “distress,” and “fever.” Treatment-related terms included “tylenol,” “anesthesia,” “cannula,” “oxygen,” “motrin,” “rituxan,” “bair hugger blanket,” and “labetalol.”

## Discussion

### Principal Findings

This study has several notable findings. First, we found that 63.33% (6053/9557) of the EHR users who recorded vital signs in flowsheets also entered at least one comment, and most of these users were either (registered) nurses or technicians. Although only 0.69% (209,055/29,995,045) of the 5 types of vital sign entries received comments, there were approximately 210,000 comments. Furthermore, our topic analysis showed that these comments mainly corresponded to how the vital signs were measured, any issues encountered while taking the vital signs, or notifications to health care providers. Further inspection of the medical terms indicated that there were still many test-, problem-, and treatment-related data that were recorded in these comments. These nurses and technicians clearly felt the need to capture more information than the simple numeric value that the flowsheet required, as documented in a study that examined the flowsheet comments for 201 patients who experienced cardiac arrest [21]. However, despite its potential usefulness, our findings suggest that there are better alternative solutions for effective information recording.

### Documentation Burden of Flowsheet Comments

First, although only a small proportion of flowsheet vital sign entries had comments, when considering a median typing speed of 30 words per minute [24], the composition of 700,000 words in 210,000 comments still implies approximately 23,333 minutes (389 hours) of comment documentation. Although, on average, each user spent about 1 minute to document, the time used for writing such comments was substantial for some Epic users because the 5 vital signs were only a small fraction of all the different types of flowsheet entries. This raises the question: Is it necessary to retain such functionality in the EHR system? Some studies have shown that patients who died tended to receive more vital sign comments than other patients [25,26]. However, it is unclear if such an association is useful in practice because it might be the severity of the condition that led to a higher volume of vital sign comments. Alternatively, it is still helpful to simplify the commenting functionality to save the users’ effort in this circumstance. According to our topic analysis, most comments were related to either notification (0.347), the context of performing a measurement (0.307), or notification (0.224). Although context might provide additional information about a measurement, it is unclear whether recording that a health care provider was notified about a measurement contributes to the understanding of a patient’s health condition. A deeper review of the identified topics could help determine the need for configuration improvements to the system. For instance, are comments related to notification entered to address potential litigation or is there a true concern for the patient’s condition? Are there other signs of patient deterioration? Are comments related to the context of the vital signs already documented elsewhere in a more appropriate location? Are comments related to the inability to take a patient’s vital signs fulfilling the nursing mantra of *if it’s not documented, it’s not done* and a potential legal consequence? Unfortunately, there has been little investigation into the motivation for recording notifications.

### Visibility of Medical Information in Comments

In addition, the medical term analysis raises another question: Should such medical information be recorded in flowsheet entry comments? In EHRs, this information should at least be recorded in clinical notes, which ensures that such information could be referred to in the future. However, flowsheet comments are not displayed or automatically pulled into any clinical notes. Rather, the only way to review an existing comment is to locate the flowsheet on the correct date and open the comment display dialog. As such, we suspect that flowsheet comments are seldom reviewed by other health care providers, except the user who made them. Although this needs to be verified (eg, which might be possible through a review of the EHR access logs), it was reported that 5.6% of the alert comments regarding potentially very important clinical safety issues were overlooked [27]. Furthermore, a study showed that only 16% of nursing notes were read by physicians and 38% were read by other nurses [28]. As such, it appears that the flowsheet entry comments might not be in a proper place to store medical-related information. Although only a fraction of notes are examined by others, recording such information in clinical notes seems a better approach to record the information and access it in the future.

### Potential Changes to EHR Design

Finally, based on this analysis, we believe there are at least two ways by which this functionality could be better oriented toward a user-centered design. First, the ability to enter free-text comments in a flowsheet row can be removed from the EHR system. Although this may save health care providers’ time and effort, it should only be considered after a careful examination of the utility of this functionality, an endeavor that is beyond the scope of this investigation. Second, we suspect that one possible explanation for recording notification is related to the potential for future lawsuits. This notion was highlighted in an interview with 5 acute care nurses, all of whom agreed that notification comments in a flowsheet help to *cover them legally* [21]. If this information must be stored, then it can be designed using structured response fields (eg, in the form of a simple checkbox) such that users do not have to click the comment entry icon, open a dialog box, and then enter comments. This design should be suitable for capturing when a measurement is reliable or unable to obtain as well. Moreover, any medical-related information should be recorded in clinical notes for future reference. We believe that such a design will be much more efficient and ensure that important information can be easily reviewed in the future. However, we acknowledge that a user-centered design approach would help understand the need for, as well as how to improve, the functionality.

### Limitation and Future Work

Despite the merits of this work, there are several limitations that we wish to highlight, which could guide future research. First, we only examined the data from a single clinical environment, which may limit the generalizability of our findings. However, this functionality exists across all Epic implementations and is therefore likely to be a widespread phenomenon. Second, we only examined vital sign comments; thus, it is unclear if these findings would hold with other types of flowsheet entries. Third, we only focused on the dominant topic when analyzing the distribution of topic groups within each vital sign. Owing to the brevity of flowsheet comments, topic modeling strategies that are explicitly oriented to handle texts of shorter length should be considered in future investigations. Fourth, we replaced only the misspellings for certain frequent terms. Correcting the spelling errors and resolving aliasing issues (ie, when 2 terms correspond to the same underlying concept) for the entire vocabulary may improve the quality of topic modeling, but determining the best approach to use is beyond the scope of this investigation. Future work may also consider extracting concepts based on specific nursing terminologies in addition to the general medical terms to interpret the comments from a nursing perspective. In addition, it might be beneficial to investigate how the use of comments varies across patient characteristics and settings (eg, comments made during a hospital encounter vs those made outside of a hospital encounter). Moreover, we only examined the comments based on their content. To fully understand this functionality and information, potential future work includes examining the motivation of recording notification in comments, the extent to which such comments would be accessed by other health care providers, and the association between the content of flowsheet comments and patients’ health-related behaviors or outcomes.

### Conclusions

Documentation burden is a recognizable issue when modern EHR systems are increasingly adopted in health care. One potential solution to reduce such burden is to simplify the existing functionalities of an EHR system. In this study, we examined the nature of vital sign comments in flowsheets using the data generated in the Epic system at VUMC. We found that most of the comments were related to the notification of a result to health care providers, the context of a measurement, and an inability to obtain a vital sign. We also extracted many medical terms (eg, symptoms or medications) from these comments. Considering that flowsheet comments are not displayed or automatically pulled into any clinical notes, we believe that such functionality can be simplified via structured response fields instead of a text input dialog to reduce health care provider effort.

## Appendix

Multimedia Appendix 1 Supplemental material.

Multimedia Appendix 2 Word cloud of medical terms regarding tests. The font size of each word is proportional to its frequency in flowsheet comments.

Multimedia Appendix 3 Word cloud of medical terms regarding problems. The font size of each word is proportional to its frequency in flowsheet comments.

Multimedia Appendix 4 Word cloud of medical terms regarding treatments. The font size of each word is proportional to its frequency in flowsheet comments.

## Abbreviations

BP: blood pressure
CLAMP: Clinical Language Annotation, Modeling, and Processing
EHR: electronic health record
LDA: latent Dirichlet allocation
Pulse: pulse rate
Resp: respiration rate
RQ: research question
SpO_2_: oxygen saturation
Temp: body temperature
VUMC: Vanderbilt University Medical Center
