# Supplementary material for: Contribution of Free-Text Comments to the Burden of Documentation: Assessment and Analysis of Vital Sign Comments in Flowsheets
Source: J Med Internet Res. 2021 Mar 4;23(3):e22806. doi: 10.2196/22806 (PMC7974764; doi:10.2196/22806)
Supplement: Multimedia Appendix 1 [file jmir_v23i3e22806_app1.docx]

Supplemental Material for “The Contribution of Free Text Comments to the Burden of Documentation: An Assessment and Analysis of Vital Signs Comments in Flowsheets”

Zhijun Yin, PhD^1,2^, Yongtai Liu, MS^2^, Allison B. McCoy, PhD^1^, Bradley A. Malin, PhD^1,2,3^, Patricia R. Sengstack, DNP, RN^1,4^

^1^Department of Biomedical Informatics, Vanderbilt University Medical Center, Nashville, TN;

^2^Department of Electronic Engineering and Computer Science, Vanderbilt University, Nashville, TN;

^3^Department of Biostatistics, Vanderbilt University Medical Center, Nashville, TN;

^4^School of Nursing, Vanderbilt University, Nashville, TN

Corresponding Author:

Zhijun Yin, PhD

2525 West End Ave. Suite 1475, Nashville, TN, 37023

Department of Biomedical Informatics, Vanderbilt University Medical Center

Email: zhijun.yin@vanderbilt.edu

The five vital signs were selected because they are commonly collected for routine clinical use in both the inpatient and outpatient settings. Table S1 shows the number of vital sign entries as well as their percentages.

Table S1. The number of entries and percentage for each vital sign recorded in Epic system at VUMC during 2018. The total number of flowsheet entries was 641,420,180.

| **Vital Sign Name** | **Count** | **Percentage** |
| --- | --- | --- |
| **Weight** | 20,355,224 | 3.17% |
| **Pulse** | 7,898,699 | 1.23% |
| **SpO2** | 7,058,507 | 1.10% |
| **Resp** | 5,994,777 | 0.93% |
| **BP** | 5,268,477 | 0.82% |
| **Heart Rate** | 4,128,970 | 0.64% |
| **Pain Score** | 4,013,674 | 0.63% |
| **Temp** | 3,774,585 | 0.59% |
| **BP Location** | 2,602,890 | 0.41% |
| **Patient Position** | 2,518,183 | 0.39% |
| **BP Method** | 1,887,918 | 0.29% |
| **IV Site Check** | 1,417,240 | 0.22% |
| **Patient Activity** | 1,396,981 | 0.22% |
| **Height** | 1,316,759 | 0.21% |
| **Alarm Parameter** | 492,570 | 0.08% |
| **Arterial Line Site Check** | 186,060 | 0.03% |
| **Head Circumference** | 47,021 | 0.01% |
| **Additional Oximeter Readings** | 2,445 | <0.01% |
| **Cuff Size** | 214 | <0.01% |

Figure S1 shows how coherence score changes as the number of topics of an LDA model varies. While the model with 22 topics (M_22_) resulted in the largest average coherence score, it can be seen that many other models had an average coherence score that is within its one standard deviation (e.g., from M_12_ to M_30_). Based on the three criteria proposed in main text, namely, relatively 1) large average coherence score, 2) small standard deviation, and 3) small number of topics, we chose model with 13 topics (M_13_) that had the largest coherence score.


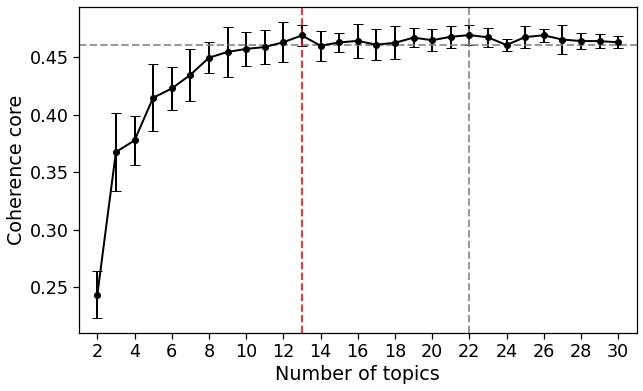


Figure S1. The average coherence score (C_v_) and its standard deviation for each candidate models. Note that square mark corresponds to the model with 22 topics (M_22_) that had the largest average coherence score. The red dashed line indicates one standard deviation below the average coherence score of M_22_.

Figure S2 shows the distribution as well as the joint distribution for the dominant and secondary topics in vital sign comments. it can be seen that many comments exhibit a dominant topic at around 0.1 and a secondary topic at around 0.075. Additionally, most comments have a limited number of words, such that we believe it is reasonable to assume that there is one dominant topic for a given comment.


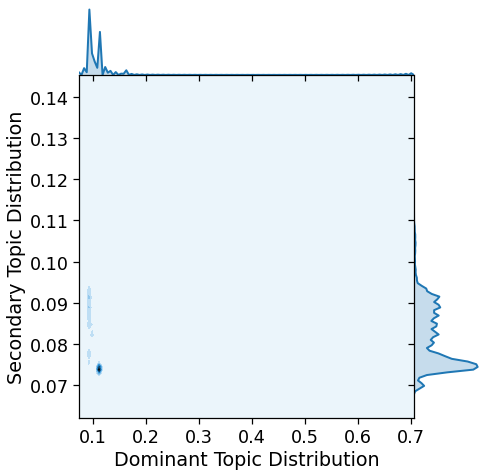


Figure S2. Joint plot of dominant topic distribution and secondary topic distribution.

The terms that were replaced due to misspelling, synonym and abbreviations are as following,

- pt -> patient
- air room -> room air
- aware -> notified
- rn notified, rn aware, notified rn -> rn notified
- nurse notified, nurse aware, notified nurse -> nurse notified
- unable to obtain -> uto
- blood pressure -> bp
- ra -> room air
- o 2, oxygen -> o2
