# Supplementary figures and images for: Contribution of Free-Text Comments to the Burden of Documentation: Assessment and Analysis of Vital Sign Comments in Flowsheets
Source: J Med Internet Res. 2021 Mar 4;23(3):e22806. doi: 10.2196/22806 (PMC7974764; doi:10.2196/22806)

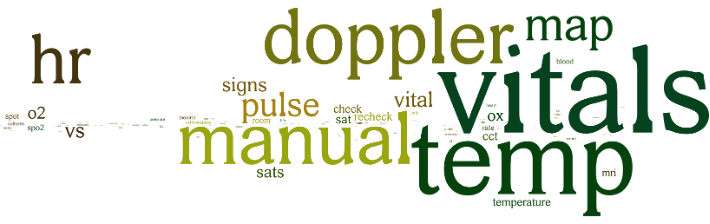

Supplement: Multimedia Appendix 2 [file jmir_v23i3e22806_app2.png]

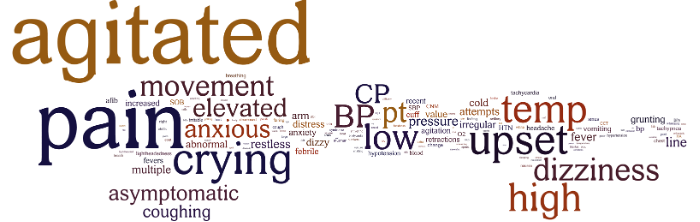

Supplement: Multimedia Appendix 3 [file jmir_v23i3e22806_app3.png]

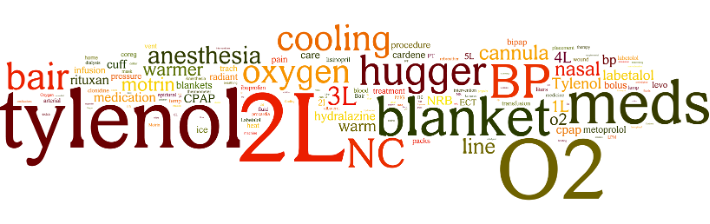

Supplement: Multimedia Appendix 4 [file jmir_v23i3e22806_app4.png]
